# SOCIOECONOMIC INEQUALITIES IN TRANSITIONS BETWEEN FRAILTY STATES IN THE VERY OLD: THE NEWCASTLE 85+ STUDY

**DOI:** 10.1093/geroni/igz038.830

**Published:** 2019-11-08

**Authors:** Carol Jagger, Nuno Mendonca, Andrew Kingston, Louise Robinson, Mohammad E Yadegarfar, Helen Hanson, Rachel Duncan

**Affiliations:** 1 Newcastle University, Newcastle upon Tyne, United Kingdom; 2 Newcastle University, Newcastle upon Tyne, England, United Kingdom; 3 University of Leeds, Leeds, England, United Kingdom; 4 Institute of Cellular Medicine, Newcastle upon Tyne, England, United Kingdom

## Abstract

Early-life socio-economic position (SEP), defined by education, remains a significant factor in disability progression in Innovation in Aging, 2019, Vol. 3, No. S1 225 GSA 2019 Annual Scientific Meeting very old age, but there is less evidence for its effect on frailty progression. We used the Newcastle 85+ Study, a longitudinal cohort of people born in 1921 and aged 85 at first assessment in 2006, and followed up at 18, 36, and 60 months. Of the 845 participants at baseline, the Fried frailty status (FFS) was available for 696 participants at baseline of whom 60% (n=414) were women. The effects of SEP in early, mid (occupation) and late-life (area deprivation) on frailty transitions between age 85 and 90 were investigated in multistate models. We found no significant effect of education on any frailty transitions. However those living in less deprived areas were less likely to die when frail (HR: 0.60, 95%CI: 0.40-0.89), and this remained significant after further adjustment for education and morbidity count.

